# Lipid peroxidation: pathophysiological and pharmacological implications in the eye

**DOI:** 10.3389/fphys.2013.00366

**Published:** 2013-12-16

**Authors:** Ya Fatou Njie-Mbye, Madhura Kulkarni-Chitnis, Catherine A. Opere, Aaron Barrett, Sunny E. Ohia

**Affiliations:** ^1^Department of Pharmaceutical Sciences, College of Pharmacy and Health Sciences, Texas Southern UniversityHouston, TX, USA; ^2^Department of Pharmacy Sciences, School of Pharmacy and Health Professions, Creighton UniversityOmaha, NE, USA

**Keywords:** lipid peroxidation, oxidative stress, peroxides, ocular diseases

## Abstract

Oxygen-derived free radicals such as hydroxyl and hydroperoxyl species have been shown to oxidize phospholipids and other membrane lipid components leading to lipid peroxidation. In the eye, lipid peroxidation has been reported to play an important role in degenerative ocular diseases (age-related macular degeneration, cataract, glaucoma, diabetic retinopathy). Indeed, ocular tissues are prone to damage from reactive oxygen species due to stress from constant exposure of the eye to sunlight, atmospheric oxygen and environmental chemicals. Furthermore, free radical catalyzed peroxidation of long chain polyunsaturated acids (LCPUFAs) such as arachidonic acid and docosahexaenoic acid leads to generation of LCPUFA metabolites including isoprostanes and neuroprostanes that may further exert pharmacological/toxicological actions in ocular tissues. Evidence from literature supports the presence of endogenous defense mechanisms against reactive oxygen species in the eye, thereby presenting new avenues for the prevention and treatment of ocular degeneration. Hydrogen peroxide (H_2_O_2_) and synthetic peroxides can exert pharmacological and toxicological effects on tissues of the anterior uvea of several mammalian species. There is evidence suggesting that the retina, especially retinal ganglion cells can exhibit unique characteristics of antioxidant defense mechanisms. In the posterior segment of the eye, H_2_O_2_ and synthetic peroxides produce an inhibitory action on glutamate release (using [^3^H]-D-aspartate as a marker), *in vitro* and on the endogenous glutamate and glycine concentrations *in vivo*. In addition to peroxides, isoprostanes can elicit both excitatory and inhibitory effects on norepinephrine (NE) release from sympathetic nerves in isolated mammalian iris ciliary bodies. Whereas isoprostanes attenuate dopamine release from mammalian neural retina, *in vitro*, these novel arachidonic acid metabolites exhibit a biphasic regulatory effect on glutamate release from retina and can regulate amino acid neurotransmitter metabolism without inducing cell death in the retina. Furthermore, there appears to be an inhibitory role for neuroprostanes in the release of excitatory amino acid neurotransmitters in mammalian retina. The ability of peroxides and metabolites of LCPUFA to alter the integrity of neurotransmitter pools provides new potential target sites and pathways for the treatment of degenerative ocular diseases.

## Introduction

Oxidative damage is involved in the pathogenesis of a variety of chronic degenerative and neurodegenerative diseases. Increasing evidence indicates that oxidative stress (OS) plays a major role in ocular pathologies including cataract, age-related macular degeneration (ARMD), glaucoma, and diabetic retinopathy (DR). Under normal physiological states, ocular tissues possess several intrinsic antioxidant enzymes to cope with OS formed as a consequence of normal metabolism. During ocular injuries, overproduction of reactive oxygen species (ROS) and free radicals overwhelms the intrinsic antioxidant mechanisms resulting in OS and ultimately development of a pathological condition. There is evidence that products of oxygen-derived free radical pathway such as peroxides and metabolites of long chain polyunsaturated fatty acids (LCPUFAs) are involved in oxidative reactions in the eye and play major roles in the pathogenesis of most ocular diseases (Greene and Paller, 1992; van Reyk et al., 2003; Shichi, 2004; Sangiovanni and Chew, 2005). LCPUFAs are essential structural components of membrane lipids that are involved in several physiological functions in the body, including central nervous system function and development, inflammation, and immunological responses in the cardiovascular system, amongst other things (De et al., 1994; Grimble, 1998; Wu and Meydani, 1998). In the eye, deficiency of docosahexaenoic acid (DHA), the most abundant LCPUFA, is associated with abnormalities in the visual system, including retinitis pigmentosa, peroxisomal disorders and compromised growth and development in infants (Sangiovanni and Chew, 2005). Tissue damage by oxygen-derived free radicals has been associated with a variety of pathological conditions in the eye. For instance, hydrogen peroxide (H_2_O_2_) a biologically derived stable oxidant intermediate, and other peroxide-induced radicals can inflict damage on ocular tissues by disrupting cellular structure and function (Wielgus and Sarna, 2008). In addition, to their patho-physiological role in the eye, oxidant-derived metabolites can exert pharmacological actions on tissues of the anterior uvea and retina. This review summarizes our current state of knowledge of the contributions of lipid peroxidation to the pathogenesis of ocular disorders as well as their pharmacological impact in the eye.

## Oxidative stress and ocular degenerative diseases

The eye is constantly exposed to radiation, atmospheric oxygen, environmental chemicals, and physical abrasion, thus, creating an environment that supports generation of elevated levels of ROS (Green, 1995; van Reyk et al., 2003). Evidence from literature supports a role for excess free radicals in ocular tissues serving as an underlying pathology in diseases such as cataracts, ARMD, glaucoma, and various forms of retinopathy (Green, 1995; Osborne et al., 1999; Shichi, 2004). The biological role of ROS in some ocular diseases is summarized below.

### Cataract

Cataract is an ocular disease that is characterized by increasing opacity of the lens resulting in visual impairment (Brennan and Kantorow, 2009; Sacca et al., 2009). Incidence of cataract increases with age and is one of the leading causes of blindness and visual impairment in elderly populations in developing countries (Li et al., 2009; Sacca et al., 2009). Aging is one of the greatest risk factors for non-congenital cataract formation, but other factors include gender, exposure to UV light, cigarette smoking, increase in ROS, decrease in antioxidant defense, and DNA damage (Brennan and Kantorow, 2009; Li et al., 2009; Sacca et al., 2009).

OS is a well-documented mechanism involved in cataract progression in which there is prevalent oxidation of lens DNA, proteins, and lipids (Sacca et al., 2009). There is evidence that ROS −•OH, O_2_• - or H_2_O_2_—can damage the lens and contribute to cataract formation (Berthoud and Beyer, 2009). Moreover, there are studies in literature showing involvement of reactive nitrogen species (RNS), such as nitric oxide (NO) in cataract formation (Varma and Hegde, 2007). Furthermore, iron has the ability to catalyze free radical reactions, thereby propagating OS. Peroxidation of lipids is another cause of cataracts formation, impairing lipid-lipid and protein-lipid interactions particularly in the lenticular fiber membranes (Sacca et al., 2009). Elevated levels of hydroperoxides, diene conjugates, and oxy-derivatives of phospholipid fatty acids have been found in human lenses and aqueous humor (AH) of patients with complicated and senile cataracts (Sacca et al., 2009). Lipid peroxidation within cells decreases glutathione (GSH) and leads to DNA impairment while lipid hydroperoxidation alters membrane permeability, structure, and microviscosity of lipid-protein mediums (Sacca et al., 2009). A study by Li et al., has reported significantly higher plasma concentrations of (Z,E) isomers of hydroxyoctadecadienoic acid (HODE), oxidation products of linoleic acid, in subjects with early cataract indicating that lipid peroxidation plays a role in the progression of lens opaqueness (Li et al., 2009).

The endogenous antioxidant defense enzymes like catalase (CAT), GSH peroxidase (GPx), superoxide dismutase (SOD), and peroxiredoxins operate to balance levels of ROS in the lens. For example, GSH neutralizes H_2_O_2_, lipid peroxides, dihydroascorbic acid, and prevents formation of disulfide bonds by keeping thiols in the reduced state. Cysteine and methionine residues also function as antioxidants through reversible protein oxidation. Thus, under normal physiological conditions, the lens is able to protect itself from OS stimuli.

### Glaucoma

Glaucoma refers to a diverse group of ocular disorders that are characterized by retinal neurodegeneration, visual field defects and blindness. Affecting approximately 60 million people globally, glaucoma is ranked as the second leading cause of irreversible blindness worldwide. This disease accounts for 9–12% of all cases of blindness in the US. Open angle glaucoma (POAG), the most prevalent form of this disease, affects about 95% of all glaucoma's encountered in US (Sommer et al., 1991). Although elevated intraocular pressure (IOP) is the major risk factor for POAG, a small population of patients exhibit visual defects with normal IOP—a condition termed normal-tension glaucoma. Thus, IOP elevation does not singularly account for the clinical manifestation of the POAG (Collaborative Normal-Tension Glaucoma Study Group, 1998; The AGIS Investigators, 2000). The etiology of POAG is attributed to multifactorial mechanisms, including OS, ischemia, vascular dysregulation, and altered NO metabolism (Sacca et al., 2007; Resch et al., 2009).

OS has been hypothesized to play a significant role in the pathogenesis of POAG (Alvarado et al., 1981, 1984). Indeed, both experimental and clinical studies implicate OS in the pathogenesis of POAG. Targets for OS include trabecular meshwork (TM) structures in the anterior segment and retinal ganglion cells (RGCs) and the optic nerve head (ONH) structures in the posterior segment of the eye. Although the exact outflow mechanism has not been completely elucidated, TM cells regulate AH outflow as part of the conventional outflow pathway and formation and turnover of extracellular matrix (ECM) (Yue, 1996). Physiological functions of both TM and Schlemm's canal decline with aging, a process that is characterized by an increase in oxidant stress (Grierson et al., 1984; Grierson and Howes, 1987). OS elicits TM degeneration and confers structural alterations in TM cytoskeleton, thereby compromising its physiological functions (Zhou et al., 1999). In support of this observation, POAG patients had higher concentrations of lipid peroxidation products in TM and AH, compared to control subjects (Babizhayev and Bunin, 1989). In the posterior segment, constant light-exposure of the LCPUFA-rich retina renders it highly susceptible to OS. Numerous OS biomarkers have been identified, including 4-hydroxy-2-nonenal (4-HNE), and malondialdehyde (MDA). Indeed, 4-HNE has been reported to induce apoptosis in primary cultures of normal ONH astrocytes in humans (Malone and Hernandez, 2007). Furthermore, MDA was elevated in vitreous and retina of ocular hypertensive rats, suggesting the presence of OS mechanisms in those tissues (Ko et al., 2005; Yucel et al., 2005).

Effective defense mechanisms against free radicals are present in biological systems. In the eye, ascorbic acid which is found in high concentrations in both the anterior and posterior segment of the eye, has been shown to be involved in ocular protection (Izzotti et al., 2006). Reduced GSH is another prominent antioxidant found in the eye that protects ocular tissues from damage caused by ROS (Riley, 1990; Costarides et al., 1991). High GSH concentrations can be found in the AH and TM of normal mammalian eyes (Kahn et al., 1983). There are findings that also suggest an antioxidant defense system in the aqueous outflow system (Ferreira et al., 2004; Yang, 2004; Gherghel et al., 2005). The retina possesses endogenous self-protective mechanisms such as the upregulation of pro-survival brain-derived neurotrophic factor (BDNF) and tyrosine kinase (TRK) receptors, expression of anti-apoptic Bcl-2 and BCL-x genes and increased expression of heat shock proteins (HSPs) (Gao et al., 1997; Levin et al., 1997; Chaudhary et al., 1999; Cui et al., 2002; Huang et al., 2007). The glial cells and ONH astrocytes are well-equipped with efficient antioxidant defense mechanism for self-protection against oxidative damage (Malone and Hernandez, 2007). These cells upregulate survival-promoting signals against ROS-induced OS and secrete neurotrophic factors that support RGC axonal regeneration (Qu et al., 2010). Clearly, endogenous defense mechanisms exist in both anterior and posterior segments to protect the eye against ROS induced damage associated with glaucoma.

### Age-related macular degeneration

ARMD is characterized by irreversible loss of central vision. It is the leading cause of vision impairment and blindness in persons aged 65 and over in industrialized countries (Ethen et al., 2007; Hollyfield et al., 2008; Hollyfield, 2010). The disease affects the macula at the center of the eye and as a consequence results in loss of central vision which significantly impacts the patient's ability to read, watch television or drive (Jarrett and Boulton, 2012) Risk factors that contribute to ARMD are heterogeneous, mainly including increasing age and different genetic predispositions, together with several environmental/epigenetic factors such as cigarette smoking, dietary habits, and phototoxic exposure. In the aging retina, free radicals and oxidized lipoproteins are considered to be major causes of tissue stress resulting in local triggers for para-inflammation, a chronic status which contributes to initiation and/or progression of ARMD (Parmeggiani et al., 2012).

Although the development of ARMD is not clearly understood, OS appears to be one feasible pathway (Winkler et al., 1999; Kasahara et al., 2005; Ethen et al., 2007; Bertram et al., 2009; Bruban et al., 2009; Hollyfield, 2010). The death of retinal pigment epithelial (RPE) cells due to OS are the primary targets in the early phase of ARMD (Kasahara et al., 2005). RPE cells are transporters of selective molecules between choroidal blood and the neural retina thereby making their death a prime factor in accompanying photoreceptor damage (Kasahara et al., 2005). Indeed, OS may cause injury to the RPE cells, the Bruch's membrane, and the choroid, which are layers in the eye involved in the pathophysiology of ARMD (Yildirim et al., 2011). OS in ocular tissues and accumulation of lipofuscin in the RPE may induce production of ROS leading to lipid peroxidation and eventual RPE cell death (Winkler et al., 1999).

Multiple defense mechanisms exist in the retina to combat OS. Endogenous antioxidants, like non-enzymatic scavengers as GSH and antioxidant enzymes such as SOD, GPx, and CAT, are the first lines of endogenous defense against OS and act by scavenging potentially damaging free radical moieties (Yildirim et al., 2011). Dietary antioxidants like the carotenoids, vitamins A, C, and E, selenium, zinc, and bioflavonoids showed decreased risk of vision loss in ARMD (Winkler et al., 1999; O'Connell et al., 2008). Clearly, opportunities exist for further investigation of preventive and therapeutic measures against ARMD that reflect the pathways of disease progression and natural defense systems.

### Diabetic retinopathy

DR is an ocular neuropathy associated with advanced forms of both diabetes types I and II (Kanwar et al., 2007; Madsen-Bouterse and Kowluru, 2008). In spite of advancement in research techniques, increased emphasis on diabetes prevention and therapeutic monitoring, DR remains an important complication of diabetes and a leading cause of blindness among young, working-age adults between 20 and 74 years (Centers for Disease Control and Prevention, 2013). Clinical appearance of DR is preceded by biochemical, molecular and hemodynamic alterations that begin early in the disease process. Both clinical and experimental data have confirmed the role of chronic hyperglycemia as the major initiating parameter in the pathogenesis of diabetes complications (Kaiser et al., 1993; Kuusisto et al., 1994; Ohkubo et al., 1995; Skyler, 1996). It appears that chronic hyperglycemia elicits a series of deleterious alterations that culminate in structural and functional damage to retina. Evidence suggests a prominent role for OS in hyperglycemia-induced alterations in DR patients and experimental animals (Yadav et al., 1997a,b; Gurler et al., 2000). While diabetes patients have elevated plasma OS biomarkers compared to normal subjects, DR patients exhibit higher concentrations of OS biomarkers than diabetes patients without DR, suggesting the significance of OS in the pathogenesis of DR (Gurler et al., 2000). It has been argued that not only does chronic hyperglycemia generate ROS, but it sets in motion several pathways including, activation of aldose reductase (sorbitol), protein kinase C (PKC), accumulation of advanced glycation-end-products (AGE) and release of inflammatory mediators and vascular endothelial-derived growth factor (VEGF) that further increases OS and concomitantly impairs endogenous antioxidant defense mechanisms (Al-Shabrawey and Smith, 2010; Cheung et al., 2010). Thus, OS is a vital parameter that links hyperglycemia and evolvement of DR in diabetic subjects (Kanwar et al., 2007; Madsen-Bouterse and Kowluru, 2008; Pan et al., 2008; Zheng and Kern, 2009).

Patients with diabetes also show increased production of lipid peroxidation, DNA damage, and protein oxidation due to OS. Indeed evidence shows elevated levels of OS biomarkers such as malondialdehyde,8-isoprostane (IsoP) E_2_, F_2_-IsoP, and lipid hydroperoxides in plasma of diabetic patients (Pan et al., 2008). Because the retina is rich in PUFAs and has increased glucose oxidation and oxygen uptake, it is susceptible to increased OS in hyperglycemia (Madsen-Bouterse and Kowluru, 2008). The observed structural and functional changes are likely to result from lipid peroxidation of the vascular endothelium.

The retina employs a complex antioxidant defense system to maintain optimal concentrations of NADPH and GSH including SOD, glucose-6-phosphate dehydrogenase, GPx, GSH reductase, and CAT (van Reyk et al., 2003; Zheng and Kern, 2009). There is evidence that DR is associated with reduced levels of GSH, SOD, CAT, and ascorbic acid (Madsen-Bouterse and Kowluru, 2008). Furthermore, there are reports that show SOD activity to be decreased in the vitreous and anterior chamber of hyperglycemic patients (Zheng and Kern, 2009).

## Role of oxidant-derived metabolites in pathogenesis of eye diseases

Stress induced by oxygen-derived radicals such as hydroxyl radical, superoxide anion and H_2_O_2_ can be deleterious to cells (Gilgun-Sherki et al., 2002; Blokhina et al., 2003). In the eye, free radical-induced tissue damage has been associated with a variety of pathological conditions. H_2_O_2_ in particular, has been reported to be involved in oxidative reactions in ocular tissues, thus, indicting a possible role for this peroxide in ocular physiology and pathology (Ohia et al., 2005). In addition to peroxides, metabolites of LCPUFA have also been implicated in the progression of ocular neurodegenerative diseases. LCPUFAs play a significant structural and physiological role within the cell. Due to the abundance of double bonds in their structure, these fatty acids are highly susceptible to lipid peroxidation to form an array of oxidant-derived metabolites. This susceptibility is further compacted in ocular tissues due to constant exposure of the eye to light and high lipid content in some ocular tissues (Mainster, 1987; Green, 1995; Organisciak et al., 1998; van Reyk et al., 2003). In spite of evidence depicting the role of OS in ocular pathology, further research is warranted to fully delineate the physiological role and biological impact of oxidant-derived metabolites in the eye. Table 1 provides a summary of oxidant–derived metabolites present in ocular tissues and their role in ocular pathology.

**Table 1:** **Summary of several Oxidant-derived metabolites and their role in ocular pathology**.

**Oxidant-derived metabolites**	**Ocular tissue presence**	**Plausible role**	**Associated ocular pathology**
**PEROXIDES**			
H_2_O_2_			Cataract, uveitis
Hydroperoxides	Lens, aqueous humor	Disrupt cell function and structure	
Lipid peroxides	Lens, retina		Cataract,DR
**LCPUFA METABOLITES**			
Hydroxyoctadecadienoic acid (HODE)	Lens	Membrane permeability alteration, cell structure changes	Cataract
4-hydroxy-2-nonenal (4-HNE)	TM, optic nerve, retina	Apoptosis, protein modification	Glaucoma, ARMD
Malondialdehyde	TM, retina, vitreous humor	Cytoskeleton alterations, functional changes	Glaucoma
ISOPs	Aqueous humor, lens, retina	Structural and functional damage to tissue	Exfoliation syndrome (XFS), cataract, DR

### Peroxides

There is evidence that H_2_O_2_ and enzymes involved in its metabolism is present in both the anterior segment and posterior segment of the eye (Rose et al., 1998; Beatty et al., 2000). H_2_O_2_ is continually produced in the AH by cellular metabolism and exposure to UV light. The concentration of H_2_O_2_ in the AH and anterior uvea in healthy persons is in the range of 30 and 70 μ M (Rose et al., 1998). In the AH and corneal epithelium, vitamin C reacts with O_2_ to form H_2_O_2_ in protecting the eye against UV radiation (Izzotti et al., 2009). In the anterior segment, free radical-induced tissue damage has been associated with a variety of pathological conditions such as cataract and experimental autoimmune uveitis (Wu et al., 1997; Shichi, 2004). In patients with cataract, concentrations of H_2_O_2_ that is ten to thirty-fold higher than healthy human subjects has been reported in lens and AH (Shichi, 2004; Wielgus and Sarna, 2008). In addition, there is evidence that lens epithelial cells are exceedingly susceptible to OS generated by H_2_O_2_. Indeed, short term exposure of epithelial cells to 25–50 μ M H_2_O_2_ will disturb normal metabolism, especially if there is a breach in antioxidant defense (Ma et al., 2004). H_2_O_2_ has also been shown to cause DNA damage in lens and corneal epithelial cells (Atilano et al., 2009). Exposure of lens epithelial cells to H_2_O_2_ causes opacification and cataract (Jin et al., 2007). The TM of the eye is particularly sensitive to H_2_O_2_-induced OS; high concentrations of H_2_O_2_ in GSH compromised eyes leads to reduced outflow in the AH (Izzotti et al., 2009).

As opposed to the anterior segment of the eye, not much is known on the role of oxygen-derived free radicals in the posterior segment of the eye. Hence further studies are needed to understand the biological role of these metabolites in ocular tissues. In the retina, H_2_O_2_ has been linked to damage due to exposure to light and oxygen (Beatty et al., 2000). Both H_2_O_2_ and enzymes that mediate its metabolism are present in the retina (Liles et al., 1991; Beatty et al., 2000; Sandbach et al., 2001). Ascorbic acid has been reported to act as an antioxidant for the removal of H_2_O_2_ in ocular tissues including the retina (Shang et al., 2003). Interestingly, the concentration of ascorbic acid in the vitreous humor has been reported to be twice that found in the AH (Rose et al., 1998). The higher levels of ascorbic acid in the vitreous humor may serve to protect the retina against the damaging effects of OS and ultraviolet radiation. The concentration of H_2_O_2_ in the vitreous humor of human patients with cataract has been reported to be two to three times more than the amount of this oxidant found in normal patients (Shichi, 2004). It remains to be determined if such high levels of H_2_O_2_ in the vitreous humor can also affect normal retinal function.

### LCPUFA peroxidation metabolites

The interaction of OS and LCPUFA metabolites is a less known area of research that could have significant implications in neuronal tissues. LCPUFA metabolites such as isoprostanes (IsoPs) and neuroprostanes (NeuroPs) have been shown to be present in mammalian tissues as well as human biological fluids (Reich et al., 2000; Cracowski et al., 2002; Fam et al., 2002; Shichi, 2004). IsoPs are spontaneously derived in abundance *in vivo* from the LCPUFA, arachidonic acid (AA). These prostaglandin (PG)-like compounds are produced by the free radical-catalyzed, non-enzymatic peroxidation of AA. Due to the fact that IsoPs are stable products, whose production increases with exposure to OS, they have gained acceptance as a reliable marker of oxidative injury in both *in vivo* and *in vitro* animal models (Gopaul et al., 1995; Delanty et al., 1997; Morrow, 2000; Shichi, 2004). Analogous to IsoPs, the NeuroPs are formed by spontaneous free-radical, non-enzymatic peroxidation of DHA and have been found to be applicable as diagnostic markers of oxidative injury (Fam et al., 2002; Shichi, 2004; Morrow, 2006). It has been reported that in the mammalian eye, oxidant stimuli can increase endogenous production of IsoPs in retina and AH (Nourooz-Zadeh and Pereira, 2000; Koliakos et al., 2003; LeDay et al., 2004; Yoshida et al., 2006; Dentchev et al., 2007). Furthermore, Koliakos et al. showed that the AH of patients with exfoliation syndrome (XFS) and cataract had an elevated level of 8-isoPGF_2α_, thus, supporting a role for IsoPs in XFS and cataract development. When used as an OS marker in ocular studies, IsoPs levels were significantly higher following tissue exposure to oxidant stress condition (Dentchev et al., 2007). To the best of our knowledge there are no reported studies on the role of neuroprotanes in ocular pathophysiology. Clearly, the need for more studies in this area of lipidomics could not be overemphasized. One can, however, speculate that since prostanoids play a role in regulation of both inflammatory and neuroprotective mechanisms in ocular tissues, both IsoPs and NeuroPs could potentially modulate disease conditions that are characterized by inflammation and neuronal degradation in the eye.

### Pharmacological implications

Although previously assumed to be inert, it is becoming apparent that oxidant-derived metabolites can exert pharmacological effects in biological tissues. Due to challenges involved in synthesizing and isolating pure metabolites and the lack of commercially available pure neuroPs and IsoPs, the pharmacological actions of these lipid metabolites have remained largely unexplored. The ability of these metabolites to exert pharmacological effects in the eye may underlie the mechanism whereby reactive oxygen metabolites play a role in ocular diseases such as glaucoma. Further studies are indeed needed to determine the exact pharmacological role of oxygen-derived free radicals in ocular diseases.

### Peroxides

The anterior segment of the eye has the capacity to protect against deleterious action of oxygen-derive free radicals and is involved in the metabolism of both endogenous and exogenous H_2_O_2_. Exogenous application of H_2_O_2_ and its synthetic peroxides have been reported to exert pharmacological/toxicological actions on tissues of the anterior uvea of several mammalian species. Earlier studies have demonstrated that intracameral injection of H_2_O_2_ in the rabbit eye can result in lowered IOP and morphological changes in the anterior chamber (Birnbaum et al., 1987; Csukas and Green, 1988). In addition, H_2_O_2_ has been shown to increase sympathetic neurotransmission in iris-ciliary bodies, an action that is dependent on the generation of reactive ROS, trace amounts of extracellular calcium and the functional integrity of mitochondrial calcium stores (Birnbaum et al., 1987; Csukas and Green, 1988; Opere and Ohia, 1998). The observed increase of sympathetic activity could lead to vasoconstriction in the anterior uvea which may cause a reduction in AH production and consequently a decrease in IOP. There is evidence that PG and thromboxanes (Tx) can mediate the excitatory effects of peroxides on sympathetic neurotransmission in mammalian irides and regulate their inhibitory effects on muscarinic receptor-induced contraction of iris smooth muscle. Graham et al. demonstrated how the cyclooxygenase (COX) inhibitor flurbiprofen attenuated the increased action of H_2_O_2_ and cumene hydroperoxide (a synthetic peroxide) on norepinephrine (NE) release (Graham et al., 2000). The enzyme CAT which is involved in the metabolism of H_2_O_2_ has also been linked to neurotransmission regulation by peroxides in ocular tissues. Matutte et al. showed that the inhibition of catalase unmasked an excitatory action of H_2_O_2_ on evoked NE release and an inhibitory effect on muscarinic receptor-induced contraction of iris smooth muscle (Matutte et al., 2000). These studies establish that inhibiting peroxide metabolism causes both sympathetic nerves and smooth muscles of the anterior uvea to be more susceptible to the harmful effects of H_2_O_2_.

The retina is highly susceptible to auto-oxidation via free radical mechanism due to its high content of polyunsaturated fatty acids. Peroxides such as H_2_O_2_, possess the ability to impair glutamate release. LeDay et al. reported that both H_2_O_2_ and cumene hydroperoxide inhibited potassium-evoked release of glutamate (using [^3^H]D-aspartate as a marker) from bovine retinae, a response prevented by the antioxidant trolox (LeDay et al., 2004). Furthermore, CAT inhibition enhanced the inhibitory action of H_2_O_2_ on glutamate release. Together these studies indicate that the inhibitory actions of peroxides on potassium-stimulated glutamate release in the retina is dependent on the generation of ROS and catalase. In a related study, these workers observed that peroxides such as H_2_O_2_ can decrease both glutamate and glycine concentrations and that catalase inhibition also caused a significant reduction in glutamate and glycine concentrations. Furthermore, LeDay et al. observed that peroxides can directly stimulate the production of COX and non-COX derived metabolites of the AA pathway and that these products are involved in mediating the effects of H_2_O_2_ on glutamanergic transmission in the retina (LeDay et al., 2004). These data indicate a vital role for endogenously produced peroxides in the regulation of amino acid neurotransmission in the retina.

### LCPUFA peroxidation metabolites

LCPUFA metabolites such as IsoPs and NeuroPs have been shown to exert pharmacological and toxicological effects in ocular tissues. Topical use of 8-iso-PGE_2_ reduces IOP in normal monkey eyes as well as those affected by glaucoma. In addition, there is evidence that IsoPs can regulate neurotransmission in human and mammalian ocular tissues. Awe et al. reported that 8-iso-PGF_2α_ enhances electrically evoked NE release from isolated human iris-ciliary bodies. This effect is blocked by the thromboxane receptor (TP) antagonist SQ-29548 suggesting that TP receptors mediate the mechanisms of 8-iso-PGF_2α_. Conversely, 8-iso-PGE_2_ was found to inhibit electrically stimulated NE release from the same tissue, a response that was not affected by SQ-29548. Taken together, these studies indicate that 8-iso-PGF_2α_ and 8-iso-PGE_2_ exert opposing effects on sympathetic neurotransmitter in human iris-ciliary bodies via different receptors or mechanisms (Awe et al., 2000). In contrast, another study by a different group showed that both 8-iso-PGF_2α_ and 8-iso-PGE_2_ enhanced electrical-evoked NE release from bovine iris, an action that was blocked by the TP receptor antagonist SQ-29548 (Awe et al., 2000; Opere et al., 2001a). These observations support the view that a species-specific difference in response to IsoPs may exist in ocular tissues.

In addition to the anterior uvea, IsoPs also regulate neurotransmission in mammalian retina. Liu et al. demonstrated that IsoPs can attenuate dopamine release from isolated bovine retina (Liu et al., 2008). There is evidence that the enzymes COX and Tx synthase may be involved in the effects of IsoPs. The COX inhibitor, indomethacin has been shown to reduce contractile response of 8-iso-PGF_2α_ in the retinal vasculature of piglets and it blocked the excitatory effect of high concentration 8-iso-PGF_2α_ on [^3^H]D-aspartate release from bovine retina (Lahaie et al., 1998; Opere et al., 2005). Studies also report IsoPs are potent vasoconstrictor of vessels in piglet retina (Lahaie et al., 1998; Hou et al., 2004). There is evidence that in mammalian retina, the pharmacological effect of IsoPs involve Tx receptor (TP) and prostanoid (EP) receptors. Opere et al. showed that 8-iso-PGF_2α_ elicited a dual effect on potassium-induced glutamate release (using [3H]D-aspartate as a marker) from bovine retina (Lahaie et al., 1998; Opere et al., 2001b; Hou et al., 2004; Opere et al., 2005, 2008). At low concentrations (1-100nM), 8-iso-PGF_2α_ activated the PGE receptors (EP) EP1/EP2 to produce an inhibitory response on glutamate release. Higher concentrations (100nM-30uM) of 8-iso-PGF_2α_ acted upon the TP receptor, promoting an excitatory effect upon glutamate release. Other studies also demonstrate that 8-iso-PGE_2_ exerts its action by activation of EP receptors because this response was blocked by EP receptor antagonists SC-19220(EP1), AH-6809(EP1-3), and AH-23848(EP4) (Zhao et al., 2008, 2009). The pharmacological actions of neuroPs remain largely unexplored but in preliminary studies, neuroPs have been shown to exert an excitatory effect on potassium-evoked glutamate release from bovine retina using [^3^H]D-aspartate as a marker. Likewise, intravitreally injected neuroP enhanced retinal glutamate and glycine levels in bovine retina (Hou et al., 2004; Opere et al., 2005, 2008; Zhao et al., 2008, 2009; Jamil et al., 2012). Glutamate is the major excitatory amino acid neurotransmitter in the retina and is involved in the transfer of visual information from the retina to the brain. A defect in glutamate synaptic transmission and uptake can result in glutamate neurotoxicity and cell death in the retina (Nucci et al., 2005; Pulido et al., 2007). Since neuronal excitotoxicity is preceded by elevation in neuronal glutamate concentrations, the ability of IsoPs and neuroPs to regulate excitatory neurotransmitter release could have significant pharmacological implications in mammalian ocular tissues.

### Perspective

Products of oxygen-derived free radical pathway such as peroxides and LCPUFA metabolites are involved in oxidative reactions in the eye and could play a major role in the pathophysiology of most ocular diseases. In addition to their pathological roles, these metabolites can exert pharmacological actions on tissues of the anterior uvea and retina. The ability of peroxides such as H_2_O_2_ to affect neurotransmitter pools from sympathetic and glutaminergic nerves in the eye may help provide insights on the underlying mechanism by which ROS contribute to ocular pathologies. It is well established that OS is an underlying pathology in ocular neurodegenerative diseases of the eye such as glaucoma and DR. Since LCPUFAs play a significant role in cellular structure and signaling, it is necessary to delineate the role of LCPUFA metabolites in the progression of ocular neurodegenerative diseases in the eye. In spite of current research, the role of lipid metabolites like NeuroPs and IsoPs in regulation of ocular disease is not yet clear. It is possible that addition of these lipid by-products to proteins could serve as diagnostic markers of endogenous peroxidation status. Furthermore, since numerous stereoisomers and regioisomers of IsoPs and neuroPs can be generated, *in vivo*, it is possible that the summation of their effects could significantly impact the homeostatic environment within the cell. Indeed further research is warranted to fully delineate the biological significance of these lipid mediators on biological systems like the eye.

The hypothesized role of oxidation in the development of ocular diseases has prompted research into the use of antioxidants as neuroprotective agents in ocular therapy. It is thought that people with low systemic antioxidant levels may be more prone to oxidative damage in ocular tissues. As such, substantial body of research, though controversial indicates that antioxidants can ameliorate certain common and uncommon ocular conditions. Despite inconclusive data suggesting that people who eat a diet rich in antioxidant vitamins such as carotenoids, vitamins C and E or minerals (selenium and zinc) are less likely to develop certain ocular diseases, several studies provide evidence supporting a role for these nutrients in preservation of vision. Indeed vitamins B, C and selenium have been shown to reduce the risk of cataract and prevent its development (Rapp et al., 2000; Chiu et al., 2007). In addition, vitamin E deficiency increases H_2_O_2_ levels and the intracellular production of ROS (Chow et al., 1999). Studies depict a synergism between vitamin E and C; vitamin C reduces oxidized vitamin E, which is crucial for protecting cell membranes from lipid peroxidation (Varma, 1991; Kang et al., 2003). Natural products such as zinc gluconate, marygold flower extract, ascorbic acid, blueberry extract and *trans*-resveratrol are also known to have antioxidant effects in the eye and have been used widely to improve vision and ocular health. Flavonoids and other antioxidants including Ginkgo biloba, Qi Ming granule have been found to be beneficial in promoting ocular health, and reduce OS in the eye (Jayle et al., 1965; Urso, 1967; Porcella et al., 2001; Rhee et al., 2001; Jarvinen et al., 2002; Majumdar and Srirangam, 2010). Furthermore, observational and experimental data from the Age-Related Eye Disease Study (AREDS) suggest that antioxidant intake may delay progression of ARMD and vision loss (Age-Related Eye Disease Study Research Group, 2001). Although the use of antioxidants looks promising for improving outcomes in ocular disorders, more investigation is warranted in order to standardize indication for use, composition and dosing for treatment. At the present time, there remains insufficient scientific data to recommend routine antioxidant intake for primary prevention of ocular neuropathies resulting from lipid peroxidation.

### Conflict of interest statement

The authors declare that the research was conducted in the absence of any commercial or financial relationships that could be construed as a potential conflict of interest.
